# Endotoxin free hyaluronan and hyaluronan fragments do not stimulate TNF-α, interleukin-12 or upregulate co-stimulatory molecules in dendritic cells or macrophages

**DOI:** 10.1038/srep36928

**Published:** 2016-11-21

**Authors:** Yifei Dong, Arif Arif, Mia Olsson, Valbona Cali, Blair Hardman, Manisha Dosanjh, Mark Lauer, Ronald J. Midura, Vincent C. Hascall, Kelly L. Brown, Pauline Johnson

**Affiliations:** 1 Department of Microbiology and Immunology, University of British Columbia, Vancouver, B.C. V6T 1Z3, Canada; 2 Department of Pediatrics, British Columbia Children’s Hospital Research Institute, Vancouver, B.C. Canada; 3 Department of Biomedical Engineering, Cleveland Clinic Lerner Research Institute, Cleveland, Ohio 44195, U.S.A

## Abstract

The extracellular matrix glycosaminoglycan, hyaluronan, has been described as a regulator of tissue inflammation, with hyaluronan fragments reported to stimulate innate immune cells. High molecular mass hyaluronan is normally present in tissues, but upon inflammation lower molecular mass fragments are generated. It is unclear if these hyaluronan fragments induce an inflammatory response or are a consequence of inflammation. In this study, mouse bone marrow derived macrophages and dendritic cells (DCs) were stimulated with various sizes of hyaluronan from different sources, fragmented hyaluronan, hyaluronidases and heavy chain modified-hyaluronan (HA-HC). Key pro-inflammatory molecules, tumour necrosis factor alpha, interleukin-1 beta, interleukin-12, CCL3, and the co-stimulatory molecules, CD40 and CD86 were measured. Only human umbilical cord hyaluronan, bovine testes and *Streptomyces hyaluronlyticus* hyaluronidase stimulated macrophages and DCs, however, these reagents were found to be contaminated with endotoxin, which was not fully removed by polymyxin B treatment. In contrast, pharmaceutical grade hyaluronan and hyaluronan fragments failed to stimulate *in vitro*-derived or *ex vivo* macrophages and DCs, and did not induce leukocyte recruitment after intratracheal instillation into mouse lungs. Hence, endotoxin-free pharmaceutical grade hyaluronan does not stimulate macrophages and DCs in our inflammatory models. These results emphasize the importance of ensuring hyaluronan preparations are endotoxin free.

Hyaluronan (HA) is a glycosaminoglycan consisting of repeating units of D-glucuronic acid and D-N-acetylglucosamine, and is an integral component of the extracellular matrix. Due to its hygroscopic and viscoelastic nature, HA can increase hydration in the interstitial matrix of tissues (reviewed in refs 1, 2 and 3). CD44 is present on most cells and is a cell surface receptor for extracellular HA^4^. At homeostasis, extracellular HA is present in its high molecular mass form (>1000 kDa), and its steady-state turnover is hypothesized to be mediated at least in part by tissue macrophages, as *in vitro* studies show the uptake and degradation of HA by alveolar macrophages^5^ and *in vivo* studies show the uptake of HA by macrophages in the developing lung^6^, in the red pulp of the spleen and in the liver^7^. High molecular mass HA (800 kDa and 2700 kDa) administered *in vitro* suppresses the pro-inflammatory response to bacterial lipopolysaccharide by U937 macrophages^8^ while inhaled HA reduces inflammatory cytokine production in a mouse model of lung cystic fibrosis^9^. Upon tissue inflammation, the size of HA at the site of damage is perturbed, as seen in mouse models of bleomycin injury^10^ and cigarette smoke exposure^11^; and the amount of HA changes, as seen in the mouse model of ovalbumin induced asthma^12^. Shorter HA fragments (<400 kDa) are reported to become damage-associated molecular pattern molecules (DAMPs) capable of inducing pro-inflammatory responses *in vitro* and *in vivo* via CD44 or TLR4, a combination of CD44 and TLR4, or TLR2 (reviewed in refs 1 and 3). Hyaluronidase (HA’se) expression is upregulated in the inflamed lung^13^ suggesting that HA turnover is increased upon inflammation. Furthermore, induced HA degradation in the skin can result in dendritic cell migration and promote allergic contact hypersensitivity^14^,^15^. These results and others have led to the model where high molecular mass HA supports homeostasis and is anti-inflammatory, whereas lower molecular mass HA and HA oligosaccharides are pro-inflammatory^16^.

However, increased HA matrices in the tissue are also associated with inflammation. HA is produced by smooth muscle cells in response to a viral mimetic, and is organized into cable-like HA structures^17^. These structures are modified by tumor necrosis factor-inducible gene 6 protein (TSG-6), a protein induced during inflammation that transfers heavy chains (HCs) from the serum proteoglycan inter-alpha-inhibitor (IαI) onto HA to form HA-HC matrices^18^,^19^. In ovalbumin induced lung inflammation, wild type mice have increased HA production^12^, and HA-HC deposition occurs in the lung tissue with extensive eosinophilia and airway hyper-responsiveness^20^. In contrast, ovalbumin treated TSG-6^−/−^ mice have reduced HA, no HA-HC deposition and decreased eosinophilia and airway hyper-responsiveness, suggesting that HA-HC complexes influence the allergic response in the lung^20^.

However, concerns regarding the pro-inflammatory effect of low molecular mass HA and HA fragments have been raised. One study stimulated macrophage cell lines with various sizes of HA but could not detect any production of the inflammatory agents, nitric oxide and tumor necrosis factor alpha (TNF-α)^21^. Another study stimulated glomerular mesangial cells with HA’se to generate HA fragments but found that endotoxin contamination was the cause of cytokine production^22^. In addition, administration of low molecular mass HA (200 kDa) or human recombinant HA’se with endotoxin free HA into a mouse skin air pouch did not stimulate an inflammatory response^23^. These results are in conflict with the idea that low molecular mass HA is pro-inflammatory and raises the possibility that HA effects may occur due to the contamination of other inflammatory molecules. Indeed, human umbilical cord HA, which has been used in the past, has both protein and nucleic acid contamination^24^, and DNA contamination in HA samples has been shown to activate human monocytic cells^25^. Given that miniscule amounts of endotoxin, for example as low as 5 pg/ml of LPS, can induce pro-inflammatory IL-6 production in mouse dendritic cells (DCs)^26^, a small amount of contamination in HA can give misleading results. Therefore, the aim of this study was to systematically test and thoroughly analyze the ability of HA preparations to stimulate a pro-inflammatory response by macrophage colony stimulating factor (CSF-1) or granulocyte macrophage colony stimulating factor (CSF-2) derived bone marrow macrophages (BMDMs) and DCs (BMDCs) *in vitro* by measuring the pro-inflammatory cytokines TNF-α, IL-1β, and IL-12, and the upregulation of CD40 and CD86 co-stimulatory molecules.

## Results

### Different sizes of pharmaceutical grade HA do not stimulate CSF-1 derived bone marrow macrophages but human umbilical cord HA does

**Figure 1:**
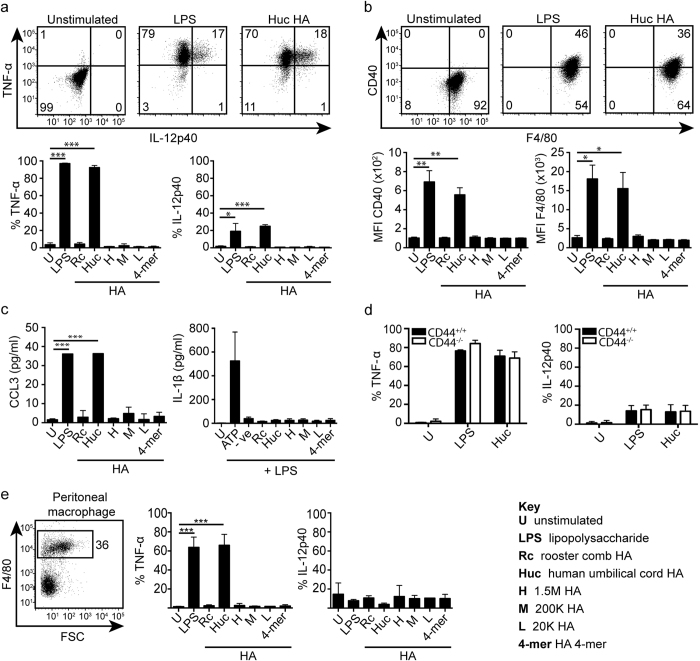
Stimulation of CSF-1 BMDMs by various HA preparations. (**a**) Representative flow cytometry plots and bar graphs summarizing the percent of cells producing intracellular TNF-α and IL-12 p40 after stimulation by various HA preparations (see Key for details). (**b**) Representative flow cytometry plots and bar graphs summarizing CD40 and F4/80 expression measured by mean fluorescence intensity units (MFI) in response to stimulation by various HA preparations. (**c**) Summary of production of CCL3 and IL-1β secreted by BMDMs after stimulation, measured by ELISA. (**d**) Bar graph summaries comparing TNF-α and IL-12 p40 production between CD44^+/+^ and CD44^−/−^ BMDMs after stimulation. (**e**) TNF-α and IL-12 p40 production from F4/80 gated peritoneal macrophages after stimulation. Graphs show an average of three mice from one experiment ± SD, repeated twice. Numbers in the flow cytometry plots refer to the percent of cells in each quadrant or box. Significance compared to the unstimulated control indicated as *p < 0.05, **p < 0.01, ***p < 0.001, unpaired student’s t-test.

In past studies, HA preparations purified from human umbilical cord or rooster comb were used that have a range of molecular masses. Low molecular mass HA (ranging from 4 to 200 kDa) derived from these preparations induced inflammatory activation of CSF-1 induced BMDMs and peritoneal macrophages (reviewed in refs 1 and 2). Now, commercial HA is purified after bacterial fermentation (Lifecore Biomedical, HTL Biotechnology) or enzymatic synthesis (Hyalose) and can generate HA of specific molecular mass ranges that are also endotoxin free. To compare the effects of HA from these different sources, CSF-1 induced BMDMs were challenged with a panel of commercially available purified HA of different specific molecular sizes (HA 1.5 M, 200 K, 20 K, <10 K daltons (see Methods for more details) from Lifecore Biomedical and HA 4-mer composed of four monosaccharides from Seikagaku), as well as HA from rooster comb (Rc), and human umbilical cord (Huc) from Sigma-Aldrich. Cells were stimulated with 100 μg/ml of HA, or 10 μg/ml of HA 4-mer, or with 100 ng/ml of ultra-pure lipopolysaccharide (LPS, from *Escherichia coli* strain 0111:B4) as the positive control. To assess the stimulatory activity of HA, key propagators of the inflammatory response were measured: the pro-inflammatory cytokines TNF-α, interleukin-12 (IL-12), and IL-1β; the co-stimulatory molecule CD40, and F4/80; and the chemokine CCL3 (MIP-1α). Only LPS and Huc HA treated macrophages produced significant amounts of TNF-α, IL-12, CCL3, and upregulated CD40 and F4/80 expression (Fig. 1a–c), measured by intracellular cytokine production and mean fluorescence intensity (MFI) using flow cytometry. The level of stimulation induced by Huc HA was similar to that of LPS. To test if the inflammatory effect of Huc HA on macrophages was dependent on the HA surface receptor CD44, CSF-1 induced BMDMs lacking CD44 expression were generated from the bone marrow of CD44^−/−^ mice and stimulated with Huc HA. CD44^−/−^ macrophages were not deficient in their pro-inflammatory cytokine production to Huc HA, demonstrating that the effect of Huc HA is not dependent on CD44 (Fig. 1d). IL-1β was not detected from cells stimulated with any of the HA after priming with LPS (Fig. 1c), suggesting that HA does not act like extracellular ATP, to activate the NLRP3 inflammasome.

Mouse CSF-1 BMDMs have similarities to *in vivo* F4/80^+^ peritoneal macrophages. Peritoneal cells were collected by lavage and stimulated by the same panel of HAs. Although these cells did not produce much intracellular IL-12 after stimulation, LPS induced substantial amounts of TNF-α, and again, this was mirrored by Huc HA (Fig. 1e).

### CSF-2 but not CSF-1 derived BMDMs and BMDCs constitutively bind FL-HA

**Figure 2:**
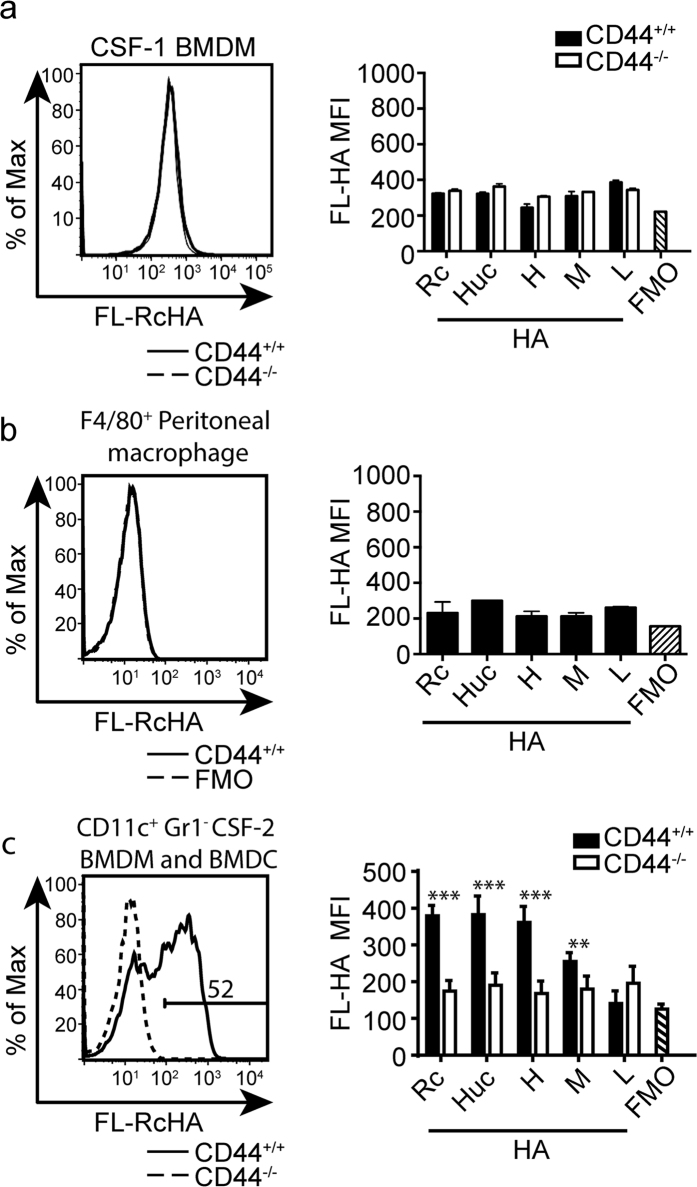
FL-HA binding by CSF-1 BMDMs, peritoneal macrophages, and CSF-2 BMDMs and BMDCs. (**a**) Representative histogram and summary graph showing the MFI of FL-Rc HA binding by CSF-1 induced BMDM. CD44^−/−^ cells (dotted line and white bar) were used as a negative control, along with a fluorescence minus one (FMO) control for FL-HA on CD44^+/+^ cells (hatched bar). (**b**) Representative histogram and graph showing the MFI of FL-Rc-HA binding by peritoneal macrophages. FMO for FL-HA on CD44^+/+^ cells (dotted line and hatched bar) is the negative control. (**c**) Representative histogram and summary graph showing the MFI of FL-HA binding by CSF-2 BMDMs and BMDCs. CD44^−/−^ cells (dotted line and white bar) were used as a negative control, along with a FMO control for FL-HA on CD44^+/+^ cells (hatched bar). The numbers in the flow cytometry plot refer to percent of cells in the gate. Key: Rc = rooster comb HA, Huc = human umbilical cord HA, H = 1.5 M, M = 200 K, L = 20 K. Graphs show an average of six mice pooled from two experiments ± SD. Significance indicated as *p < 0.05, **p < 0.01, ***p < 0.001, unpaired student’s t-test.

To determine if this lack of response could be due to the inability of CSF-1 BMDMs to bind HA, we evaluated HA binding using fluorescein labelled-HA (FL-HA). We had previously shown that prior to stimulation, BMDMs and peritoneal macrophages do not bind appreciable levels of HA^27^. Here we confirmed that no appreciable level of binding was seen in CSF-1 BMDMs or peritoneal macrophages by flow cytometry with FL-HA from Huc, Rc, and specific sizes of HA (Fig. 2a,b). In contrast, CSF-2 elicited bone marrow derived cultures contain both CD11c^+^ HA binding and non-binding populations, which have been attributed to HA binding BMDMs and non-HA binding BMDCs, respectively^28^. Figure 2c shows that a significant percentage of the CSF-2 cultured cells bound FL-HA. CD44^+/+^ CSF-2 BMDMs bound significant levels of Rc FL-HA, Huc FL-HA, 1.5 M FL-HA, and 200 K FL-HA compared to CD44^−/−^ CSF-2 BMDMs. However, specific binding of 20 K FL-HA to CD44 was not detectable, but this and the lower labelling with the 200 K FL-HA may be attributed to their lower fluorescein conjugation ratio. Given the ability of a significant percentage of these cells to bind FL-HA, we next determined if the different sized HA samples could generate an inflammatory response.

### Different sizes of pharmaceutical grade HA do not simulate CSF-2 derived BMDMs and BMDCs, but human umbilical cord HA does

**Figure 3:**
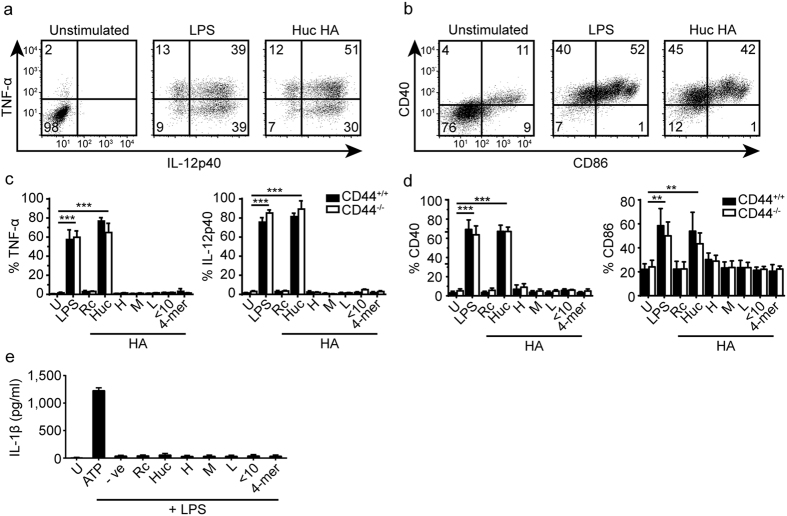
Stimulation of CSF-2 BMDMs and BMDCs by various HA preparations. (**a**) Representative flow cytometry plots showing the percent of CD44^+/+^ CSF-2 BMDMs and BMDCs positive for intracellular TNF-α and IL-12 p40 production after stimulation. (**b**) Representative flow cytometry plot showing the percent of CD40 and CD86 expressing cells after stimulation with various HA preparations. (**c**) Bar graphs summarizing TNF-α and IL-12 production from CD44^+/+^ and CD44^−/−^ BMDMs and BMDCs after HA stimulation. (**d**) Bar graphs summarizing the percent of CD40 and CD86 expressing cells in CD44^+/+^ and CD44^−/−^ cells after HA stimulation. (**e**) IL-1β production from CD44^+/+^ CSF-2 BMDMs and BMDCs after priming with 10 ng/ml LPS then stimulation with various HA. Numbers in the flow cytometry plots are the percent of cells in each quadrant. Key: U = unstimulated, −ve = media only, LPS = lipopolysaccharide, Rc = rooster comb HA, Huc = human umbilical cord HA, H = 1.5 M, M = 200 K, L = 20 K. Graphs show an average of six mice pooled from two experiments ± SD. Significance compared to the unstimulated control indicated as *p < 0.05, **p < 0.01, ***p < 0.001, unpaired student’s t-test.

*In vitro* stimulation of CSF-2 BMDMs and BMDCs with the panel of HA samples (HA 1.5 M, 200 K, 20 K, <10 K, 4-mer, Rc and Huc HA) demonstrated that only Huc HA led to significant levels of TNF-α and IL-12 being produced (Fig. 3a,c). Similarly, no responses were obtained with pharmaceutical grade HA of 40 K, 200 K and 1.5 M molecular mass from HTL Biotechnology. Only Huc HA treatment significantly upregulated CD40 and CD86 co-stimulatory molecules, and these elevated levels were comparable to those of LPS stimulation (Fig. 3b,d). CD44^−/−^ cells also produced TNF-α and IL-12, and increased co-stimulatory molecule expression to the same degree as CD44^+/+^ cells demonstrating a CD44 independent effect (Fig. 3c,d). Again, IL-1β production was not detected from CSF-2 BMDMs and BMDCs primed with LPS and then stimulated with any of the HA for 24 hr (Fig. 3e).

### Different sizes of pharmaceutical grade HA do not stimulate *ex vivo* splenic macrophages or DCs while human umbilical cord HA does

**Figure 4:**
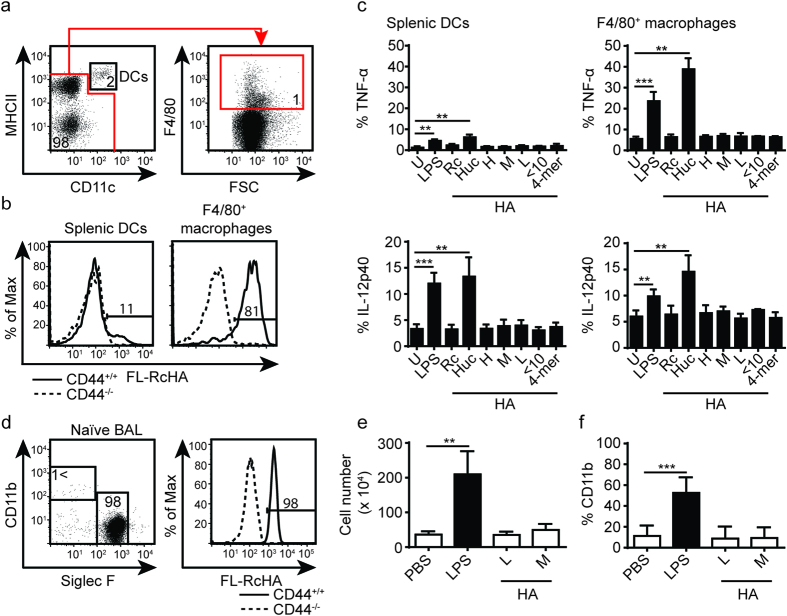
*In vitro* stimulation of splenic macrophage and DCs by various HA preparations and the intratracheal instillation of HA into the lung. (**a**) Gating strategy for analyzing splenic F4/80^+^ CD11c^low^ macrophages (box 1) and CD11c^+^ MHCII^hi^ DCs (box 2). (**b**) Representative histograms showing FL-Rc-HA binding by CD44^+/+^ and CD44^−/−^ splenic DCs and macrophages. (**c**) Bar gaphs summarizing percent of cells positive for intracellular TNF-α and IL-12 p40 production by splenic DCs and macrophages after stimulation with various HA. (**d**) Representative flow cytometry plots showing gating strategy to identify leukocytes and alveolar macrophages from the BAL. Alveolar macrophages are Siglec F^+^ CD11c^+^ and HA binding, while infiltrated leukocytes are CD11b^+^. (**e**) Total number of cells in the BAL and the percent of CD11b^+^ cells after 24 hr following intratracheal instillation with PBS, LPS, 20 K (L), or 200 K (M) HA. Numbers in the flow cytometry plots refer to the percent of cells in the box or gate. Key: U = unstimulated, LPS = lipopolysaccharide, Rc = rooster comb HA, Huc = human umbilical cord HA, H = 1.5 M, M = 200 K, L = 20 K. Graphs show an average of six mice pooled from two experiments ± SD. Significance compared to the unstimulated control indicated as *p < 0.05, **p < 0.01, ***p < 0.001, unpaired student’s t-test.

To test if the effect of HA was recapitulated with analogous *ex vivo* cells, splenic macrophages and DCs were tested for their ability to produce pro-inflammatory cytokines. Splenic macrophages were identified as CD11c^low^ F4/80^+^ cells, whereas splenic DCs were identified as CD11c^high^ MHCII^high^ cells (Fig. 4a). The HA binding of splenic F4/80^+^ macrophages and DCs was measured by FL-HA and was similar to the CSF-2 BMDMs and BMDCs: the majority of F4/80^+^ macrophages bound HA whereas the majority of splenic DCs did not (Fig. 4b). When the splenic cells were stimulated with the panel of HA molecules, again only Huc HA stimulated a significant percentage of cells to produce TNF-α and IL-12 (Fig. 4c).

Thus, pharmaceutical grade HA (1.5 M, 200 K, 40 K, 20 K, <10 K, 4-mer) and Rc HA were not pro-inflammatory for *in vitro* derived or *ex vivo* macrophages and DCs. Huc HA was the only HA that stimulated cytokine production and co-stimulatory molecule upregulation to similar levels seen with LPS stimulation, and this occurred independently of CD44.

### Administration of 20 K and 200 K HA *in vivo* does not induce lung inflammation

Previous studies show that HA is upregulated in the lung in response to oxidative damage, and intratracheal instillation of short chain HA increases lung airway hyper-responsiveness^29^. Low molecular mass HA is increased in cigarette smoke sensitized lung^11^ and induces chemokine production in *ex vivo* alveolar macrophages^30^. Similar to CSF-2 derived macrophages and splenic F4/80^+^ macrophages, CD11b^−^ Siglec F^+^ alveolar macrophages isolated by bronchoalveolar lavage (BAL) bind high levels of FL-RcHA (Fig. 4d). To test if smaller sized HA can cause inflammation in the lung, 100 μg of 200 K HA or 20 K HA, PBS, or 25 μg LPS were introduced separately into mouse lungs via intratracheal instillation. At steady-state, the alveolar airspace predominantly contains alveolar macrophages, but upon inflammatory stimulation such as with LPS, CD11b^+^ leukocytes are recruited to the alveolar space^31^. Thus, the degree of lung inflammation was measured after 24 hr by comparing the percentage of CD11b^+^ leukocyte infiltration into the alveolar airspace and the total number of cells retrieved from the BAL. LPS treated mice had significantly increased numbers of total cells and percentage of CD11b^+^ leukocytes, whereas the cell numbers in both 200 K and 20 K HA treated mice were similar to PBS treated mice (Fig. 4e,f). Thus, no evidence was found to support the ability of these sizes of pharmaceutical grade HA to stimulate an *in vivo* inflammatory response leading to leukocyte recruitment in the lung.

### Fragmented HA is not inflammatory to CSF-2 derived BMDMs and BMDCs

**Figure 5:**
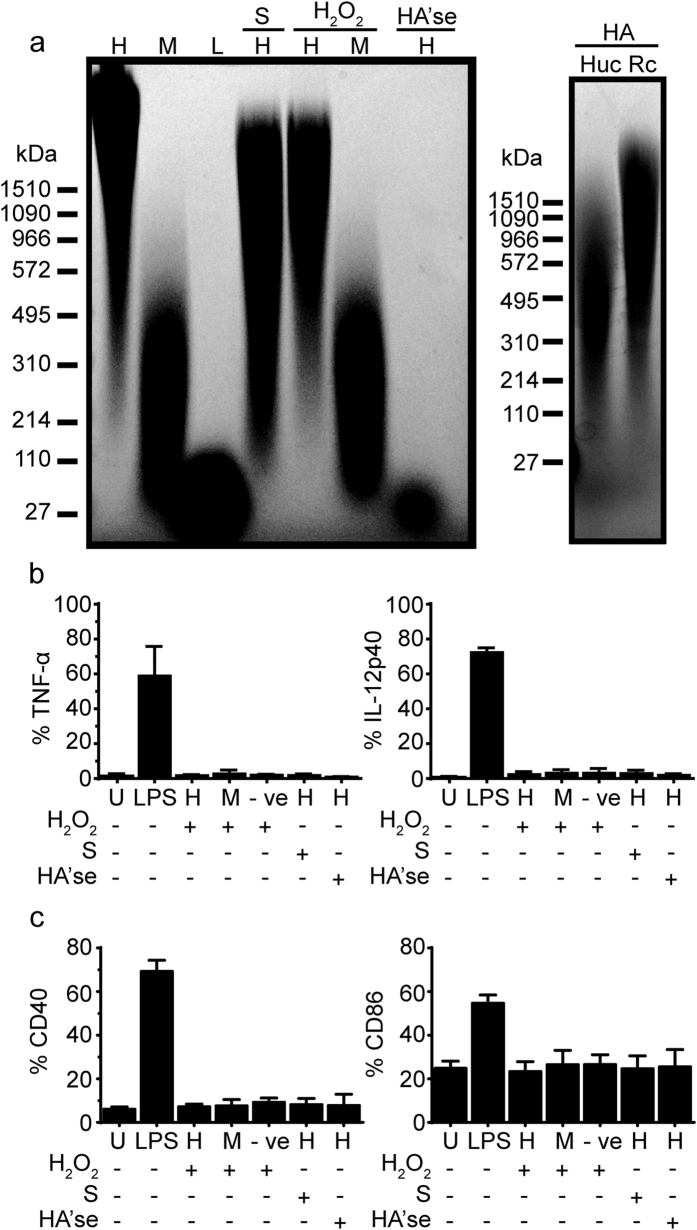
Sizes of various HA preparations and their effect on CSF-2 BMDMs and BMDCs. (**a**) Representative agarose electrophoresis gel showing the size range of various HA preparations from Lifecore or fragmented by overnight sonication (S), H_2_O_2_ incubation, or Bov HA’se digestion. H = 1.5 M, M = 200 K, L = 20 K. The gel on the right shows Rc and Huc HA. (**b**) Summary of the percent of cells producing TNF-α and IL-12 p40 after stimulation with the above HA preparations. (**c**) Summary of the percent of cells expressing CD40 and CD86 in response to HA. Graphs show an average of six mice pooled from two experiments ± SD. Significance compared to the unstimulated control indicated as *p < 0.05, **p < 0.01, ***p < 0.001, unpaired student’s t-test.

One could argue that *in vivo* generated HA fragments may differ from purified HA available from commercial sources both by how the fragments are generated (enzymatic or chemical cleavage) and how HA is associated with other proteins. Tissue HA can be fragmented enzymatically^11^ or by reactive oxygen species^32^ potentially generating altered HA structures that may be detectable by sentinel immune cells. To test whether immune cells are activated by HA fragments generated by these methods, CSF-2 derived BMDMs and BMDCs were stimulated with HA degraded physically by sonication, chemically via hydrogen peroxide (H_2_O_2_), or enzymatically using bovine testes (Bov) HA’se. These methods generated a range of HA sizes (Fig. 5a). However, stimulating cells with these prepared HA fragments did not elicit any significant TNF-α or IL-12 production (Fig. 5b) and did not upregulate co-stimulatory molecule expression (Fig. 5c).

### HC-HA complexes do not induce an inflammatory response in CSF-2 BMDMs and BMDCs

**Figure 6:**
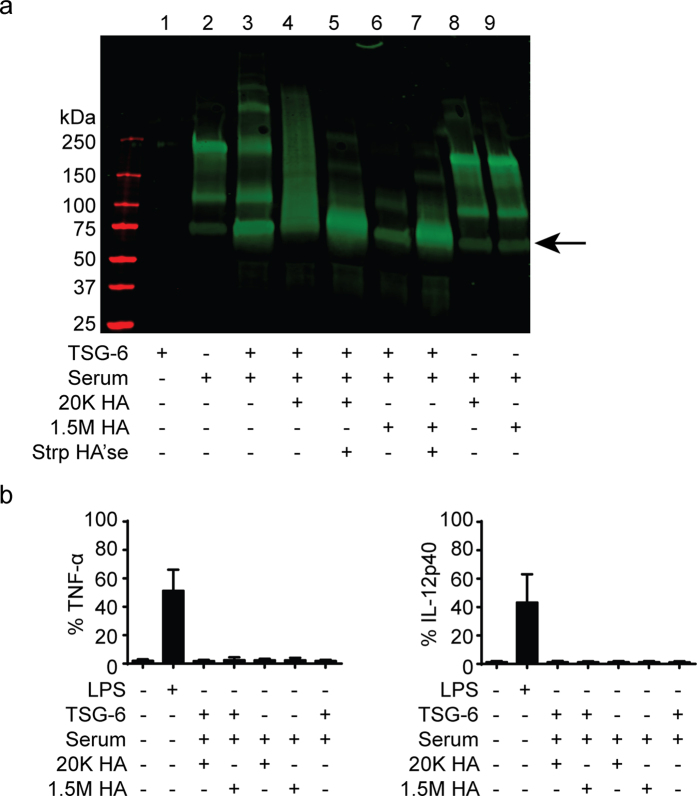
Western blot demonstrating the generation of HA-HC complexes and their effect on stimulating CSF-2 BMDMs and BMDCs. (**a**) Representative anti-IαI (HC) Western blot showing the HC of IαI in serum and when bound to HA by TSG-6 and when released by Strp HA’se. (**b**) Summary graphs of the percent of cells expressing TNF-α and IL-12 p40 production after stimulation with *in vitro* generated HA-HC complexes or with control samples containing just serum and TSG-6. Graphs show an average of six mice pooled from two experiments ± SD. Significance compared to the unstimulated control indicated as *p < 0.05, **p < 0.01, ***p < 0.001, unpaired student’s t-test.

As the formation and deposition of TSG-6 mediated HA-HC complexes occurs *in vivo*, for example during ovalbumin induced lung asthma in mice^20^, HA-HC may be another form of HA that initiates the inflammatory response in macrophages or DCs. HA-HC complexes were generated *in vitro* by incubating mouse serum as a source of IαI, mouse recombinant TSG-6, and 1.5 M or 20 K HA at 37 °C. The presence of HA-HC complexes was confirmed after HA’se treatment by Western blot using an IαI specific antibody (Fig. 6a). In the serum, the HC of IαI is covalently associated with bikunin generating higher molecular mass forms of ~100 (preIαI) and 200 (IαI) kDa^19^. When TSG-6 adds HC to HA, the size will depend on the size of the HA and how many HC are conjugated: lane 4 shows a range of molecular weight for HC conjugated to 20 K HA; lane 6 shows a band that did not enter the gel when HC was conjugated to 1.5 M HA. Treatment of these samples with HA’se releases the HC from the HA and results in an increase in the HC band at 75 kDa (see arrow, lanes 5 and 7). This demonstrates that TSG-6 covalently bound HC to both 20 K and 1.5 M HA. These unpurified reaction mixtures containing the HA-HC complexes as well as recombinant TSG-6, serum and any unconjugated HA, were then used to stimulate CSF-2 BMDMs and BMDCs at a final concentration of HA of 10 μg/ml. However, no TNF-α or IL-12 was detected (Fig. 6b), showing that neither the *in vitro* derived 20 K HA-HC nor the 1.5 M HA-HC complexes initiated pro-inflammatory cytokine production in CSF-2 derived BMDMs or BMDCs.

### Huc HA and HA’se are contaminated with endotoxin

**Figure 7:**
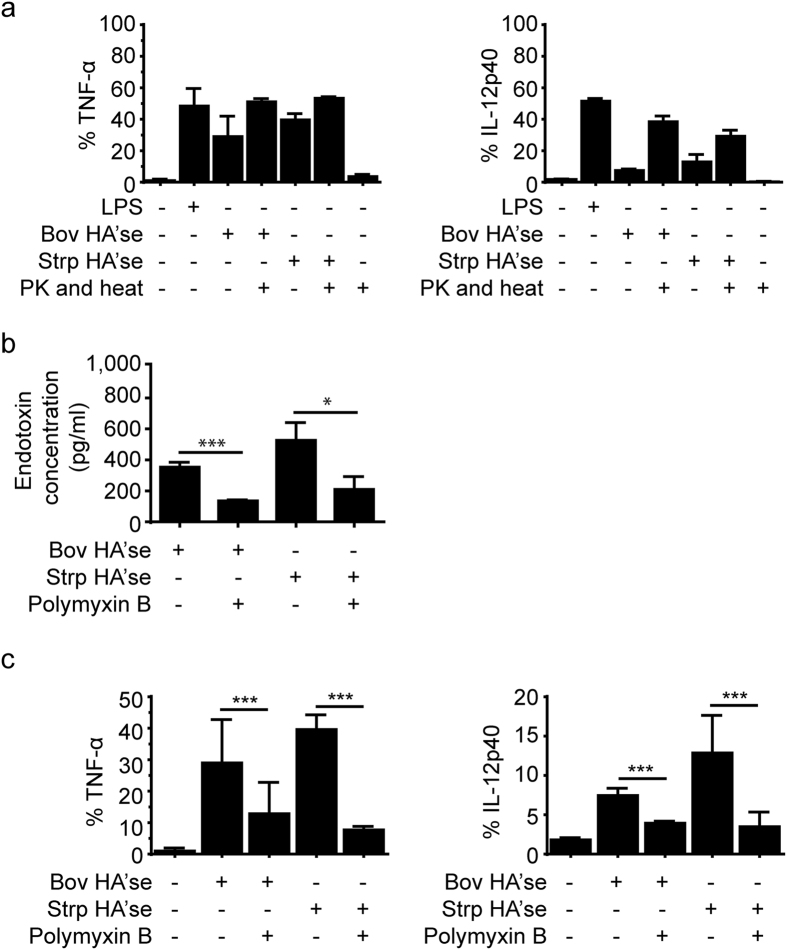
The effect of Bov and Strp HA’se on the activation of CSF-2 BMDMs and BMDCs. (**a**) Bar graphs summarizing the percent of cells producing TNF-α and IL-12 p40 after exposure of the cells to Bov and Strp HA’se stimulation for 8 hr; proteinase K (PK) and heat was used to degrade HA’se. (**b**) Concentration of endotoxin present in 20 U/ml of Bov and Strp HA’se before or after polymyxin B treatment as measured by the LAL assay. (**c**) Bar graphs summarizing the percent of TNF-α and IL-12 p40 producing cells assessed by intracellular cytokine labelling after stimulation with HA’ses treated with or without polymyxin B. Graphs show an average of six mice pooled from two experiments ± SD. Significance compared to the unstimulated control indicated as *p < 0.05, **p < 0.01, ***p < 0.001, unpaired student’s t-test.

Previous studies have used HA’se to generate lower molecular mass HA to stimulate cells *in vitro*. Bovine (Bov) or *Streptomyces hyaluronlyticus* (Strp) HA’ses were added to CSF-2 induced BMDMs and BMDCs, and these enzymes induced TNF-α and IL-12 production (Fig. 7a). However, HA’ses degraded with proteinase K and then denatured by heat inactivation also stimulated cytokine production, demonstrating that the ability of these reagents to stimulate macrophages and DCs was independent of the HA’se protein. The presence of endotoxin contamination was confirmed in both Bov and Strp HA’ses by the Limulus Amebocyte Lysate (LAL) assay^33^ (Fig. 7b). Treatment with agarose beads coupled to polymyxin B, a cationic neutralizer of LPS^34^, was able to significantly lower endotoxin levels (Fig. 7b), and this caused significantly less TNF-α and IL-12 production (Fig. 7c), suggesting that the explanation for the positive effect was endotoxin contamination.

**Figure 8:**
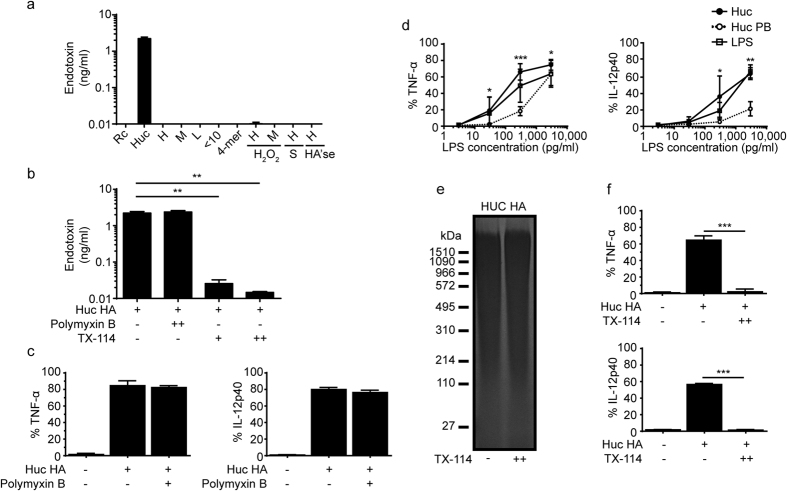
Endotoxin contamination and removal from Huc HA and its effect on the production of pro-inflammatory cytokines by CSF-2 BMDMs and BMDCs. (**a**) Level of endotoxin contamination in 100 μg/ml of the HA preparations measured by LAL assay. (**b**) Endotoxin concentration in Huc HA before and after one (+) or two (++) treatments with polymyxin B or Triton-X 114 (TX), measured using the LAL assay. (**c**) Percent of Huc HA stimulated cells making TNF-α and IL-12 p40 as assessed by intracellular labelling after polymyxin B treatment. (**d**) Graphs showing the percent of cells making TNF-α and IL-12 p40, assessed after intracellular labelling, after stimulating the cells with decreasing amounts of LPS, Huc HA or Huc HA treated with polymyxin B (PB). The amount of LPS present in the Huc HA sample was as indicated and compared to an equivalent amount of LPS. The effectiveness of polymixin B on reducing the Huc HA response is shown. (**e**) Representative agarose electrophoresis gel showing the amount of Huc HA present before and after two rounds of TX-114 treatment. (**f**) Percent of cells making TNF-α and IL-12 p40 production after stimulation with Huc HA or Huc HA after two cycles of TX-114 treatment. Graphs show an average of six mice pooled from two experiments ± SD. Significance indicated as *p < 0.05, **p<0.01, ***p < 0.001, unpaired t-test.

The presence of endotoxin in Bov and Strp HA’se raised the possibility that the inflammatory properties of Huc HA may also be caused by contamination. The same concentration of various sized HA and HA fragments used in this study was tested in the LAL assay, revealing large amounts of endotoxin contamination in Huc HA (approximately 2 ng/ml in the 100 μg/ml of Huc HA used to stimulate cells), but no detectable levels of endotoxin were found in all other purified forms of HA (Fig. 8a). Although previous studies have used polymyxin B to remove endotoxin from HA samples^35^,^36^, the efficacy of this method of endotoxin removal was not determined. One study showed polymyxin B was only effective when removing endotoxin from low Huc HA concentrations^37^. Here, we found that polymyxin B treatment was not effective in removing endotoxin contamination from Huc HA at 100 μg/ml (Fig. 8b), nor was it effective at reducing the pro-inflammatory effect of Huc HA, used at 100 μg/ml (Fig. 8c). However, a titration comparing Huc HA and LPS at equivalent concentrations as the contamination (3000 pg/ml, 300 pg/ml, 30 pg/ml, and 3 pg/ml LPS) showed that contaminated Huc HA and LPS have a similar ability to stimulate cytokine production upon dilution, whereas the titration of Huc HA treated with polymyxin B revealed a limited but significant reduction in amount of TNF-α and IL-12 production, especially at the lower pg/ml concentrations (Fig. 8d). Polymyxin B was able to remove small amounts (pg/ml) of endotoxin from the Huc HA sample, making the more diluted samples incapable of activating cells. Interestingly, IL-12 was not produced in response to lower concentrations of LPS whereas the cells were still able to produce TNF-α. As an alternative method for endotoxin removal, Huc HA was treated with two cycles of Triton X-114. This method uses micellar phase separation to remove LPS endotoxin from the aqueous phase^38^. Huc HA treated for two Triton X-114 cycles significantly removed most of the endotoxin, leaving levels just at the point of detection by the LAL assay at 10 pg/ml, over a 100 times reduction (Fig. 8b). Importantly, two cycles of Triton X-114 treatment did not significantly change the amount of Huc HA recovered in the aqueous phase (Fig. 8e), and Huc HA treated with two Triton X-114 cycles significantly reduced TNF-α and IL-12 production to background levels (Fig. 8f) demonstrating that the pro-inflammatory effect of Huc HA is due to endotoxin (LPS) contamination.

Taken together, we conclude that 1.5 M, 200 K, 40 K, 20 K, <10 K, 4-mer, Rc, *in vitro* fragmented HA, as well as *in vitro* generated HA-HC complexes do not stimulate macrophages or DCs to make the key pro-inflammatory cytokines, TNF-α and IL-12. The stimulatory effect of Huc HA was attributed to endotoxin contamination and occurred independently of CD44 on macrophages and DCs.

## Discussion

This work re-evaluates the ability of various HA fragments below 250 kDa to act as DAMPs and concludes that pharmaceutical grade HA and HA fragments by themselves do not stimulate macrophages or DCs to produce TNF-α, IL-1β, IL-12, or CCL3, and do not upregulate CD40 or CD86 co-stimulatory molecules. Here, both *in vitro* derived and *ex vivo* macrophages and DCs were treated with pharmaceutical grade HA of different molecular mass, with chemically and enzymatically generated HA fragments, and with *in vitro* generated HA-HC complexes, and none of the above pro-inflammatory responses were observed. In the context of these results, this raises the possibility that observed increases in lower molecular mass HA in inflamed tissues may be a consequence, not a cause, of a type 1 inflammatory response where TNF-α, IL-1β, and IL-12 are produced and the co-stimulatory molecules, CD40 and CD86, are upregulated. In this study, we cannot exclude that some other proinflammatory cytokine or chemokine could be made in response to HA, but if this was the case, we would have expected to see leukocyte recruitment in response to HA instilled into the lungs. However, a full transcriptional and proteomic profile will be required to definitely determine if HA or HA fragments can activate any aspect of a pro-inflammatory response in macrophages and DCs.

In contrast to the purified HA and HA fragments from Lifecore, Huc HA and HA’se treated samples induced a CD44 independent pro-inflammatory response that was subsequently shown to be due to endotoxin contamination. Notably, polymyxin B, a cationic neutralizing agent of LPS^34^ was able to remove endotoxin from the HA’se protein, but was much less efficient at removing endotoxin from Huc HA preparations. Perhaps the negatively charged HA molecules^39^ can interfere with the binding of cationic polymyxin B to LPS, which is also negatively charged^40^. Alternatively, both HA and LPS contain N-acetylglucosamine, which is important for binding to polymyxin^41^, therefore excess HA may compete with LPS for binding to polymyxin B. It is thus imperative to monitor the removal of endotoxin, ideally to 1–3 pg/ml levels, to minimize macrophage and DC activation. We found Triton X-114 to be more effective in removing endotoxin from HA preparations. Because of this potential endotoxin contamination in certain HA preparations and HA’ses, researchers should review the sources and methods in which past studies utilized and generated HA fragments. Some information in this regard can be found in two recent reviews^1^,^2^. Furthermore, researchers should always check that the endotoxin levels of HA reagents are 1–10 pg/ml or lower.

Past studies used HA to stimulate cells at various concentrations ranging from 10 μg/ml to 1 mg/ml^21^,^30^,^42^,^43^. In this study, a concentration of HA (100 μg/ml) was used to stimulate cells, and no response was observed. Other experiments with 1.5 M, 200 K, 20 K and Rc HA at concentrations as high as 500 μg/ml and HA 4-mer concentrations of 50 μg/ml also did not elicit an inflammatory response.

Several forms of fragmented HA were used to stimulate cells *in vitro* in an attempt to cover the possible forms of HA that might be encountered *in vivo*. Various sizes and differently generated fragments as well as complexed HA-HC were used, all with no effect. Pharmaceutical grade HA is purified from fermenting *Streptococcus equi* under specific conditions, such that the rate of HA polymerisation and availability of substrates allow for the generation of HA in a specific size range^44^. Both *S. equi* and vertebrate hyaluronan synthases (HAS) are Class 1 HAS^44^, and bacterial HA synthases are not known to generate a HA molecule that is different from mammalian HA. It is also unlikely that the purification procedure introduces subtle changes in this HA as it remains capable of binding avidly to CD44 even after conjugation with fluorescein^27^,^28^. *In vivo*, the form of HA present at both homeostasis and during inflammation is not clear; it could be nascent, or complexed with other HA binding proteins or matrix components. It is also unclear whether HA generated *in vivo* is inflammatory or possesses a different conformational structure that can be recognized by cells. While the addition of 200 K and 20 K sizes of pharmaceutical grade HA did not induce inflammatory leukocyte infiltration into the alveolar space *in vivo*, we cannot exclude the possibility that complexed forms of HA generated *in vivo* in response to an infection or damage are differentially recognized by pattern recognition receptors. For example, it is not known how well the HA-HC complexes generated *in vitro* mimic HA-HC complexes generated *in vivo* in response to an inflammatory insult. However, the *in vitro* data presented here suggest that high molecular mass HA is not inflammatory, and smaller HA fragments, on their own, are insufficient to trigger an inflammatory response.

The conflicting roles of HA being anti-inflammatory and pro-inflammatory depending on its size and form has always been difficult to understand mechanistically. However, as studies demonstrating a direct effect of HA on inflammation have relied on stimulating cells or animals with exogenous HA^29^,^30^,^37^,^43^,^45^,^46^,^47^, caution is needed, and efforts should be made to ensure that the effects are not due to picogram levels of endotoxin contamination, from either the HA source or from the HA’se used to generate the fragments. Endotoxin contaminated Huc HA tested in this study activated macrophages and DCs regardless of the HA binding ability of CD44 or CD44 expression, consistent with the ability of LPS to induce pro-inflammatory effects through Toll-like receptor 4 (TLR-4), which was also reported to be necessary for HA induced inflammation^43^,^48^.

*In vivo* mouse models where low molecular mass or fragmented HA are generated without introducing exogenous HA such as from the overexpression of human hyaluronidase 1^14^ may provide a better approach. In this case, expression of human hyaluronidase 1 in the skin did not lead to spontaneous inflammation. However, its induction enhanced DC migration to the draining lymph node, and this enhanced contact hypersensitivity if induced concurrently with a sensitizing agent^14^. Irrespective of whether HA is a cause or consequence of inflammation, HA is increased upon inflammation, and its abundance and/or size in the tissue, and possibly the serum, may provide a useful biomarker for the state of inflammation. This is an ongoing area of research, for example in arthritis^49^, breast cancer metastasis^50^, and liver fibrosis^51^.

In conclusion, purified, endotoxin-free, exogenously added HA of various molecular mass, fragmented HA, *in vitro* complexed HA-HC, and HA’ses were unable to induce pro-inflammatory responses in *in vitro* derived and *ex vivo* murine macrophages and DCs, as measured by the production of TNF-α, IL-1β, IL-12, CCL3, and the expression of the co-stimulatory molecules, CD40 and CD86. HA fragments of 200 K and 20 K did not stimulate the *in vivo* pro-inflammatory response of leukocyte recruitment after direct lung instillation. Endotoxin contamination was present in Huc HA, Bov HA’se, and Strp HA’se, and was responsible for their pro-inflammatory ability as this was lost upon endotoxin removal. This demonstrates the need to ensure HA preparations and HA’se treated samples are free of pg amounts of endotoxin contamination.

## Methods

### Animals

C57BL/6 J mice were from Jackson Laboratories, CD44^−/−^ mice^52^ were backcrossed onto the C57BL/6 J background for nine generations, and maintained at the University of British Columbia (UBC). Six to ten week old mice were used for experiments. All animal experiments were conducted in accordance with the Canadian Council of Animal Care Guidelines using protocols approved by the University of British Columbia Animal Care Committee.

### Reagents

Pharmaceutical grade HA of various sizes (HA15M-1 (~1680 kDa), HA200K-1 (~234 kDa), HA20K-1 (~28 kDa), HA5K-1 (<10 kDa) were purchased from Lifecore Biomedical and are referred to as 1.5 M, 200 K and 20 K HA. The HA 4-mer (tetrasaccharide) was from Seikagaku. Rc HA (H5388) and Huc HA (H1504) were from Sigma-Aldrich. Fluorescein-labelled HA (FL-HA) was made by the Antibody Laboratory at UBC or according to ref. 53. Ultra-pure LPS was from *Escherichia coli* strain 0111:B4 from InvivoGen. Bovine testes HA’se was from Sigma-Aldrich (H4272), and HA’se isolated from *Streptomyces hyaluronlyticus* was from EMD Millipore (389561). Proteinase K was from Fisher BioReagents and saponin from Calbiochem. J558L cell-conditioned medium was the source of CSF-2^54^, L929 cell-conditioned medium was the source of CSF-1. Mouse recombinant TSG-6 was from R&D Systems (2326-TS-050). All other reagents and chemicals were from Sigma-Aldrich.

### Antibodies

F4/80 (BM8) phycoerythrin-cyanin 7 (PE-Cy7), CD44 (IM7) Pacific Blue, CD11b (M1/70) Pacific Blue, CD11c (N418) PE-Cy7, Gr1 (RB6-8C5) Pacific Blue, CD86 (P03.1) phycoerythrin (PE), MHC II (M5/114.15.2) allophycocyanin (APC), Ly6C (HK1.4) peridinin chlorophyll protein-cyanin 5.5 (Percp-Cy5.5), Ly6G (1A8) PE/Cy7, Siglec-F (E50-2440) PE, CD40 (HM40-3) fluorescein isothiocyanate (FITC), CD86 (GL1) PE, TNF-α (MP6-XT22) PerCP-eFluor 710, and IL-12/23p40 (C17.8) PE antibodies and streptavidin PE were purchased from eBioscience, Biolegend, or the Antibody Laboratory at UBC. The Dako anti-IαI antibody was from the Programs of Excellence in Glycosciences at the Cleveland Clinic.

### Cell isolation and culture

CSF-2 BMDM and BMDC and CSF-1 BMDM were prepared as described previously^27^,^28^. Briefly, bone marrow cells were isolated from the femurs and tibias of mice and treated with RBC lysis buffer (0.84% ammonium chloride, 2 mM Tris-Cl pH 7.2) for 5 min at RT. Two million bone marrow cells were cultured in a 100 × 15-mm petri dish (Falcon) in 10 ml complete RPMI 1640 medium (10% fetal bovine serum (FBS) 20 mM Hepes, 1× nonessential amino acid, 55 μM 2-mercaptoethanol, 50 U/ml penicillin/streptomycin, 1 mM sodium pyruvate (all from Invitrogen), 2 mM L-glutamine, (Sigma-Aldrich) supplemented with J558L supernatant containing 20 ng/ml of CSF-2). Non-adherent cells were harvested on day 7. For CSF-1 derived BMDMs, 1.5 × 10^7^ bone marrow cells were cultured in 10 ml of DMEM, 20% FBS, 1 mM sodium pyruvate, 2 mM L-glutamine, 50 U/ml penicillin/streptomycin, and 8% L929 cell-conditioned media (LCCM) as a source of CSF-1. BMDMs were harvested using Versene at day 5 and re-plated in a 96-well plate overnight, and cells stimulated on day 6. Peritoneal macrophages were obtained by peritoneal lavage using Ca^2+^ and Mg^2+^ free Hanks’ balanced salt solution (Invitrogen). Recovered cells were plated in DMEM with 20% FBS in a flat bottom 96-well plate for 2 hr, non-adherent cells discarded, and the remaining adherent cells (enriched for peritoneal macrophages) used for analysis. Splenic cells were isolated from spleen homogenized by collagenase digestion (1 mg/ml collagenase IV (Worthington) in PBS and 5% FBS) for 20 min at RT.

### Cell staining

CSF-2 BMDM and BMDC cell suspensions were incubated in 2.4G2 tissue culture supernatant to block Fc receptors. Cells were then incubated with fluorescently labelled antibodies for 20 minutes at 4 °C, followed by two washes of 300 μl 2% bovine serum albumin (BSA), 2 mM EDTA, in PBS. For measurement of intracellular cytokines, cells were stimulated in complete medium for 8 hr at 37 °C, with 10 μg/ml Brefeldin A (Sigma) added after 2 hr to block protein transport. Cells were then labelled with antibodies as above, fixed in 4% paraformaldehyde (ThermoFisher), permeabilized in saponin buffer (0.1% saponin, 1% BSA in PBS), and labelled with antibodies specific to TNF-α or IL-12, for 30 minutes at RT. Brefeldin A was not used for flow cytometry of co-stimulatory molecules, and the cells were stimulated for 24 hr. Antibody labelled cells were analyzed by flow cytometry (BD LSRII).

### Intratracheal instillation and bronchoalveolar lavage (BAL)

Mice were anesthetized by isoflurane and instilled with 50 μl of PBS containing LPS (500 μg/ml), 200 K HA (2 mg/ml) or 20 K HA (2 mg/ml). Mice were euthanized 24 hr later by isoflurane overdose, and alveolar cells harvested by BAL via catheterization of the trachea and washing three times, each with 1 ml 1× PBS, 2% BSA, and 2 mM EDTA. Cells were pelleted and then treated with RBC lysis buffer.

### Cell stimulation and analysis of inflammation

Splenocytes, peritoneal macrophages, BMDMs or BMDCs were stimulated in 96-well, non-tissue culture treated, round bottom plates (Falcon). BMDMs were left to adhere overnight prior to stimulation (and peritoneal macrophages for 2 hr). Cells were cultured at 2 × 10^5^ cells per well in 200 μl complete medium lacking CSF-1 or -2 and stimulated with 20 ng LPS (InvivoGen) or 20 μg of various HA preparations, with the exception of the HA 4-mer, where 2 μg was added. Alternatively, cells were stimulated with 100 μl of the reaction mixture containing 10 μg of HA complexed to HC in a final volume of 200 μl media. Cells were stimulated for 8 hr for intracellular cytokine measurement or 24 hr for ELISA (TNF-α, IL-12, IL-1β and CCL3, eBioscience) and co-stimulatory molecule expression. To measure IL-1β secretion, cells were primed with 2 ng of LPS in 200 μl media, and then 3 hr later, either 5 mM ATP or 20 μg of various HA preparations were added.

### Fragmentation of HA

1.5 M HA at 2 mg/ml was digested with 2 U/ml Bov HA’se overnight at 37 °C to produce HA fragments. One ml of 4 mg/ml 1.5 M HA in a microcentrifuge tube was sonicated in an ultrasonic bath (VWR) operating at 35 kHz for 24 hr. Two mg/ml of 1.5 M and 200 K HA were treated with 100 μM H_2_O_2_ for 24 hr at 37 °C to generate fragmented HA.

### Inactivation of HA’ses

Bov and Strp HA’ses at 200 U/ml were degraded with 100 μg/ml proteinase K in 50 mM Tris pH 7.5, 5 mM CaCl_2_ for 30 min at 50 °C then the enzyme denatured at 95 °C for 5 min.

### Polymyxin B removal of LPS

One ml of Huc HA at 200 μg/ml was incubated with 200 μl of packed polymyxin B coupled agarose beads (Sigma) in microcentrifuge tubes, and mixed end-over-end for 1 hr at RT. The supernatant was removed and treated a second time with the beads. 100 μl of the treated Huc HA was added to 100 μl of cells in complete media for stimulation.

### Triton X-114 removal of LPS

Essentially as described in ref. 38: 5 μl (1%) of cold Triton X-114 was added to 500 μl of ice-cold 2 mg/ml HA preparation and vortexed gently for 5 min. The mixture was then transferred to 37 °C for 5 min to allow micelles to form and aggregate. Samples were then centrifuged at 10,000× g for 30 seconds and kept at 37 °C until the Triton X-114 formed a clear, immiscible layer at the bottom of the tube. The top aqueous fraction was collected. This cycle was repeated once more, and then used to stimulate cells. Endotoxin levels were measured by Endpoint Chromogenic limulus amebocyte lysate assay (Lonza).

### Visualization of HA on agarose gel

HA (2–4 μg) was loaded on a 1% agarose (Fisher) gel in 40 mM Tris, 20 mM acetic acid, 1 mM EDTA (TAE buffer) and run at 100 V for approximately 1.5 hr. The gel was stained overnight in 0.005% Stains-All (Sigma) in 50% ethanol filtered twice, then de-stained in 10% ethanol and imaged on a gel documentation light cabinet (Alpha Innotech Corp).

### Generation of HA-HC complexes *in vitro*

HA-HC complexes were generated as previously described^19^. Briefly, 1.5 M or 20 K HA (100 μg) was incubated with mouse serum (100 μl), and mouse recombinant TSG-6 (12.5 μg) in 1 ml of RPMI 1640 for 4 hr at 37 °C. The generation of HA-HC complexes was verified, and then 100 μl of the reaction mixture, containing the HA-HC complexes, recombinant TSG-6 and mouse serum, was used to stimulate cells.

### Detection of HA-HC complexes with Western blot

HA-HC complexes generated above (10 μl) and control samples were incubated or not with Strp HA’se (2 μl) in PBS to a total of 25 μl for 1 hr at 37 °C. Each sample (10 μl) was heated to 95 °C for 5 mins and loaded on to a polyacrylamide gel, transferred to a membrane, blotted with the primary anti-IαI antibody (Cleveland Clinic) followed by a LI-COR donkey anti-rabbit secondary antibody for HC detection using an Odyssey Scanner.

### Data analysis

Flow cytometry was analysed using Flowjo VX (Treestar). Graphs were generated using Graphpad Prism 6. Data shown are the average ± standard deviation (SD). Significance was determined by a Student two-tailed, unpaired t test with *p < 0.05, **p < 0.01, ***p < 0.001, unless otherwise specified.

## Additional Information

**How to cite this article**: Dong, Y. *et al*. Endotoxin free hyaluronan and hyaluronan fragments do not stimulate TNF-α, interleukin-12 or upregulate co-stimulatory molecules in dendritic cells or macrophages. *Sci. Rep.* **6**, 36928; doi: 10.1038/srep36928 (2016).

**Publisher's note:** Springer Nature remains neutral with regard to jurisdictional claims in published maps and institutional affiliations.
